# NAA80 bi-allelic missense variants result in high-frequency hearing loss, muscle weakness and developmental delay

**DOI:** 10.1093/braincomms/fcab256

**Published:** 2021-10-26

**Authors:** Irena J J Muffels, Elsa Wiame, Sabine A Fuchs, Maarten P G Massink, Holger Rehmann, Jiska L I Musch, Gijs Van Haaften, Didier Vertommen, Emile van Schaftingen, Peter M van Hasselt

**Affiliations:** 1 Department of Metabolic Diseases, Division of Pediatrics, Wilhelmina Children’s Hospital University Medical Centre Utrecht, Utrecht University, 3584 EA Utrecht, the Netherlands; 2 Laboratoire de biologie moléculaire, UCLouvain-Cliniques Universitaires Saint-Luc, 1200 Brussels, Belgium; 3 Department of Genetics, Section of Genome Diagnostics, Division Laboratories, Pharmacy and Biomedical Genetics, 3584 CX Utrecht, the Netherlands; 4 Department of Energy and Biotechnology, Flensburg University of Applied Sciences, 24943 Flensburg, Germany; 5 Department of Genetics, Division Laboratories, Pharmacy and Biomedical Genetics, 3584 CX Utrecht, the Netherlands; 6 Mass Spectrometry Platform, de Duve Institute, UCLouvain, 1200 Brussels, Belgium; 7 Laboratory of Physiological Chemistry, De Duve Institute, UCLouvain, 1200 Brussels, Belgium

**Keywords:** actin acetylation, actin dynamics, post-translational actin modifications, Baraitser-Winter, hearing loss

## Abstract

The recent identification of NAA80/NAT6 as the enzyme that acetylates actins generated new insight into the process of post-translational actin modifications; however, the role of NAA80 in human physiology and pathology has not been clarified yet. We report two individuals from a single family harbouring a homozygous c.389T>C, p.(Leu130Pro) *NAA80* genetic variant. Both individuals show progressive high-frequency sensorineural hearing loss, craniofacial dysmorphisms, developmental delay and mild proximal and axial muscle weakness. Based on the molecular structure, we predicted and confirmed the *NAA80* c.389T>C, p.(Leu130Pro) variant to result in protein destabilization, causing severely decreased NAA80 protein availability. Concurrently, individuals exhibited a ∼50% decrease of actin acetylation. NAA80 individual derived fibroblasts and peripheral blood mononuclear cells showed increased migration, increased filopodia counts and increased levels of polymerized actin, in agreement with previous observations in *NAA80* knock-out cells. Furthermore, the significant clinical overlap between NAA80 individuals and individuals with pathogenic variants in several actin subtypes reflects the general importance of controlled actin dynamics for the inner ear, brain and muscle. Taken together, we describe a new syndrome, caused by NAA80 genetic variants leading to decreased actin acetylation and disrupted associated molecular functions. Our work suggests a crucial role for NAA80-mediated actin dynamics in neuronal health, muscle health and hearing.

## Teaser

Muffels et al. report two brothers harbouring a homozygous missense NAA80 genetic variant, with sensorineural hearing loss, craniofacial dysmorphisms, developmental delay and muscle weakness. At the cellular level, they show that the NAA80 variant leads to NAA80 deficiency, causing decreased actin acetylation and disrupted actin cytoskeletal functions.

## Graphical Abstract

**Graphical Abstract:**
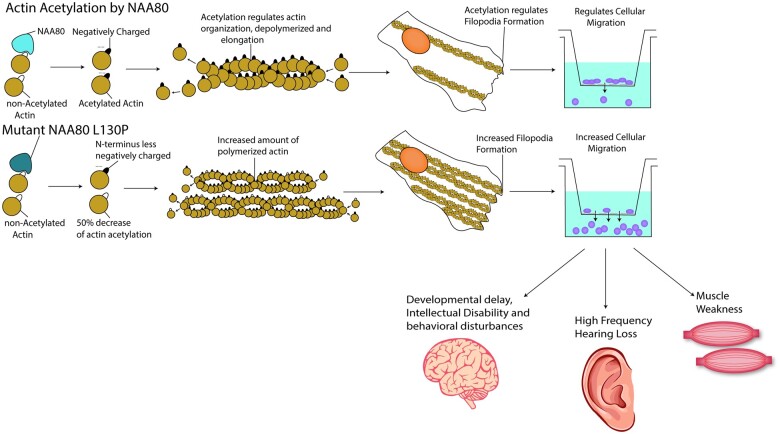

## Introduction

Actins are among the most conserved and ubiquitously expressed proteins in vertebrates. In humans, six actin genes exist; four actins are primarily involved in muscle contraction, whereas β-actin (ACTB) and γ-1-actin (ACTG1) are cytosolic and facilitate cellular movement, exocytosis, organelle trafficking and regulation of genetic transcription.^1–3^ In muscle cells, actins and myosins form tightly organized myofibrils that facilitate muscle contraction,^4^ whereas in non-muscle cells, the actin network is highly dynamic, resulting in dynamic modelling of actin filaments that fit the cells’ particular needs.^5^^,^^6^

Actins differ mostly at their N-terminus, which consists of three or four acidic residues (glutamate or aspartate), the first of which is acetylated on its amino group to suppress the positive charge of the amine. The acetyltransferase involved in this modification is NAA80, that belongs to a family of N-acetyltransferases, modifying the N-terminus of a wide range of proteins.^7–11^ NAA80 is specific for actin as a result of the untypically highly acidic N-terminus of actins. In fact, no other N-acetyltransferase can compensate for loss of NAA80.^7^^,^^8^^,^^10^ The specificity of NAA80 for actin is not only due to the acidic character of the N-terminus of actins, but also due to other specific interactions that NAA80 can establish with actins. These include formation of a ternary complex with Profilin-2 (PFN2) , a protein known to bind to monomeric actin.^7^^,^^12^ In most cell types, 99% of the N-termini of all actin subtypes are acetylated.^8^ At a cellular level, the increased negative charge density induced by actin acetylation is thought to result in altered actin dynamics, including actin stability and elongation, that influence cell size, filopodia formation and cellular migration.^9^^,^^13^ For example, *NAA80* knockout cell lines showed increased filopodia counts, increased cell size and increased cellular migration.^9^ However, consequences of absent actin acetylation have only been studied in immortalized cell lines, which may be more tolerant to actin cytoskeletal dysfunction than well-differentiated tissues. As a result, the importance of actin acetylation in organisms has yet to be determined.

Here, we describe two brothers, homozygous for a c.389T>C, p.(Leu130Pro) variant in *NAA80,* with high-frequency sensorineural hearing loss and craniofacial dysmorphisms. We found that the *NAA80* variants impacted protein stability, resulting in decreased NAA80 protein levels and decreased actin N-terminal acetylation. At a cellular level, changes in filopodia formation, actin polymerization and motility were observed. The brothers with NAA80 genetic variants have significant phenotypic overlap with patients harbouring γ-actin and β-actin mutations, suggesting that the disrupted actin dynamics are causing the NAA80 phenotype. Together, these results implicate an important role for actin acetylation in human physiology and pathology.

## Materials and methods

### Ethics

Informed consent was obtained from the parents of probands 1.2 and 1.4 to collect residual material collected for diagnostic purposes to include in the Wilhelmina Children’s Hospital metabolic biobank (TCBio 19-489/B, https://tcbio.umcutrecht.nl). From proband 1.2, we included both fibroblasts and PBMCs. From proband 1.4 we were only able to include PBMCs. In the same biobank, we included residual material of four different paediatric healthy fibroblast lines. Healthy adult donor PBMCs (*N* = 6) were obtained by using the Minidonor Service, an ethics review board approved blood donation facility at the UMC Utrecht (protocol number 18-774). Since healthy donor material availability was limited, we were not able to match individuals exactly by age and gender with healthy donors. Additionally, informed consent was obtained for publication of facial images and medical images of both individuals. All procedures performed in studies involving human participants were in accordance with the ethical standards of the institutional and/or national research committee(s) and with the Helsinki Declaration (as revised in 2013).

### Whole-exome sequencing and genetic analyses

Exomes were enriched using Agilent SureSelect XT Human All Exon kit V5 and sequenced on a HiSeq sequencing system (Illumina). Reads were aligned to hg19 using Burrows–Wheeler Aligner. Variants were called using Genome Analysis Toolkit Variant Caller and annotated, filtered and prioritized using the Bench NGS Lab platform (Agilent-Cartagenia, Leuven, Belgium) and/or an in-house designed ‘variant interface’ and manual curation. The minimal coverage of the full target was 15× 94.2%. All common polymorphisms with a minor allele frequency >0.25 were filtered out using several public databases including 1000 genomes database,^14^ Ensembl GRCh37 genome browser,^15^ exome aggregation consortium database,^16^ genome aggregation database (gnomAD)^17^ and database of single-nucleotide polymorphisms. All variants with one of the following effects were included: Splice-site, STARTLOSS, STOP, Frameshift, STOPLOSS, NONSYNONYMOUS. Variant calling was performed using the complete human reference genome (hg19, NCBI release GRCh37). Sanger sequencing was performed to identify if the same genetic variants were present in other probands, using the following primers: for NAA80: 5′-GATGCTTGTGCTGACCTCA-3′ and 3′-GGGCTGGTTCAGCACC-5′, for PLXNB1: 5′-CCCCTGGCTCCACAGGGTCGC-3′ and 3′-GCTCACACTCGACCCGGGCC-5′, for HDAC6: 5′-AACTATGACCTCAACCGGCCA-3′ and 3′-CTGTGAACCAACATCAGCTCT-5′, for PRR14L: 5′-TCTAGCCTGGCATGGTGG-3′ and 3′-GCTGGAGATTCACATCAATCATTT-5′. Individuals were evaluated according to the Wilhelmina Children’s hospital clinical practice, and all laboratory analyses were performed in ISO 9001/ISO15189 accredited diagnostic laboratories.

### Structural assessment of variants

A sequence-based search of the pdb database (12 December 2020) identified the entries 6NAS, 6NBE, 6NBW and 5WJD as informative. To assess putative structural consequences of the variants, the identified structural data were inspected and individual structures superimposed in Bragi.^18^ Graphical representations were generated based on pdb entry 6NBE using MolScript^19^ and Raster3D.^20^

### NAA80 expression in *Escherichia coli* and in HAP1 cells

Recombinant NAA80 expression and purification from *Escherichiacoli* were performed as previously described.^8^ Phosphorylated primers 5′-CTGCCAAGCCCCCACCCCACACTTG-3′ and 5′-CATCAGGCAGAGGGGGAAGGCATCTG-3′ were used to introduce the c.389T>C, p.(Leu130Pro) variant in *NAA80* sequence cloned in pET-22b and the construct was validated by sequencing. To generate lentiviral constructs for the expression of wild-type and mutant NAA80 in NAA80 knockout HAP1 cells, the NAA80 open reading frame (with and without the c.389T>C, p.(Leu130Pro) variant) was inserted in the XbaI and BsrGI sites of the vectors pUB81, pUB82 and pUB83. These vectors are derivatives of the pLVX-PURO (Clontech, Mountain View, USA) driving expression of the gene of interest under the control of EF-1alpha, SV40 and CMV promoter.^21^ Human Embryonic Kidney 293T cells were transiently co-transfected with these lentiviral vectors and second generation packaging plasmids psPAX2 and pMD2 with Jet-Pei reagent. Forty-eight hours after transfection, NAA80 knockout HAP1 cells were infected as described previously and selected with 2 µg/ml puromycin.^8^

### Wild-type NAA80 cDNA transduction in fibroblasts

NAA80 individual and healthy control fibroblasts were transduced by combining 8 µg/µl Polybrene (#TR-1003-G, Bio-connect, Huissen, The Netherlands) with 100 µl of lentiviral vectors with second generation packaging plasmids. Selection was performed using 2 µg/ml puromycin.

### Western blot

Procedures for western blot in HAP1 knockout cells were described previously.^8^ For the fibroblast western blots, cells were harvested with 0.05% trypsin/EDTA, washed once with Phosphate Buffered Saline (PBS) without calcium and magnesium and lysed in Laemmli buffer (SDS, Glycerol, Tris) supplemented with a protease inhibitor cocktail (#P8340, Sigma Aldrich, Darmstadt, Germany). Protein concentration was determined using the Bicinchoninic acid assay kit, using bovine serum albumin as standard (#23225, Thermo Fisher Scientific, Waltham, USA). Blotting was performed using wet transfer (1.5 hours, 100 Volt) in blotting buffer (20% methanol, Sodium Dodecyl Sulfate (SDS)/glycine ). The following antibodies were used: anti‐NAA80 (#15476‐1; Proteintech, Rosemont, USA) and β-tubulin (CST2128S, Cell Signaling Technology, Danvers, USA).

### Mass spectrometry analysis of actin N-termini

A mass spectrometry approach was used to quantify actin N-terminal acetylation precisely. As acetylation of a peptide changes the intensity of the signal obtained by mass spectrometry, we converted *in vitro* non-acetylated actin to fully acetylated actin by incubation with labelled ^13^C_2_, D_3_-acetyl-CoA and recombinant NAA80. Trypsin digestion and mass spectrometry allowed the quantification of the non-labelled N-terminal peptide (i.e. the endogenously acetylated) and the labelled N-terminal peptide (i.e. unacetylated in intact cells).

In practice, ^13^C_2_, D_3_-acetyl-CoA was synthesized from acetic anhydride-^13^C_4_, D_6_ (Sigma-Aldrich) and coenzyme A (sodium salt; from Sigma-Aldrich) as previously described.^22^ Fibroblasts (from one confluent 10-cm plate), PBMC (from 9 ml of fresh blood) or buffy coat cells (from 9 ml fresh blood) were resuspended in a buffer containing 25 mM HEPES, pH 7.4, 0.5 mM phenylmethylsulfonyl fluoride, 5 mg/l leupeptin and 5 mg/l antipain, submitted to two cycles of freezing in liquid nitrogen and thawing, and lysed by vortex-mixing. Cell extracts were centrifuged for 15 min at 15,000*g* and 4°C. Protein concentration was determined with the Bradford assay using γ-globulin as a standard. Fifty micrograms of cell extract supernatants were incubated at 37°C, in a mixture (100 µl) containing 50 mM Tris, pH 7.5, 25 mM Kalium Chloride, 1 mM MgCl_2_ and 0.15 mM ^13^C_2_, D_3_-acetyl-CoA. The reaction was initiated by the addition of 150 ng recombinant NAA80^8^ and stopped after 5 min with 400 µl cold methanol and 100 µl chloroform.

For mass spectrometry analysis, samples were treated essentially as described.^8^ Separation was performed with a linear gradient of 4–50% solvent B (0.1% fluoroacetic acid in 98% acetonitrile) for 100 min, 50–75% solvent B for 10 min and holding at 95% for the last 5 minutes at a constant flow rate of 300 nl/min. Intact peptides were detected in the Orbitrap at a resolution of 120 000. Peptides were selected for tandem mass spectometry (MS/MS) using high energy collision dissociation setting at 35; ion fragments were detected in the Orbitrap at a resolution of 50 000. A targeted mass list was included for the six theoretical peptide sequences (see Supplementary Table 1), considering oxidation of Met, acetylation of the N-terminus, incorporation of 2 ^13^C and 3 deuterium and charge states +2. The electrospray voltage applied was 2.1 kV. MS1 spectra were obtained with an automatic gain control target of 4 × 10^5^ ions and a maximum injection time of 50 ms, and targeted-MS2 spectra were acquired with an automatic gain control target of 2 × 10^5^ ions and a variable injection time. For MS scans, the *m*/*z* scan range was 350–1800. The resulting MS/MS data were processed and quantified using Skyline (version 19.1.0.193).^23^

Trypsin was specified as cleavage enzyme allowing up to two missed cleavages, four modifications per peptide and two charges. Mass error was set to 10 ppm for precursor ions and 0.05 Da for fragment ions. An example of analysis is illustrated in Supplementary Fig. 1.

### Filopodia count

Fibroblasts of the NAA80 proband 1.2 were cultured in Ham F12 medium (#11765054, Thermo Fisher) supplemented with 10% fetal bovine serum (#F7524, Sigma Aldrich), 100 UI/ml penicillin and 100 μg/ml streptomycin, in a humidified incubator at 37°C and 5% CO_2_. Cells were split at 80% confluency. For the assay, cells were plated in a 96-well plate (500 cells/well). After 24 hours, cells were fixed with 4% formaldehyde and permeabilized with 0.1% Triton X-100. Cells were incubated with phalloidin-tetramethylrhodamine B isothiocyanate (TRITC) (#P1951, Sigma-Aldrich, 1:100) and mounted with Hoechst (#B2261, Sigma 1:2000). Cells were visualized with the Leica TCS SP8 (Wetzlar, Germany) using 63X enlargement. Pictures were analysed with FiJi ImageJ software, using the FiloQuant package.^24^ For analysis, GraphPad Prism was used (Version 8.0.0 for Windows, GraphPad Software, San Diego, USA).

### Flow cytometry analysis

Actin polymerization was determined by staining PBMCs and fibroblasts with Phalloidin-TRITC and subsequent analysis by flow cytometry. For fibroblasts, cells were plated in a 6-well plate (5 × 10^4^ cells/well) and grown until 80% confluency. Cells were harvested using TrypLE (#12604013, ThermoFisher), washed and immediately fixed and permeabilized. Afterwards, cells were incubated Phalloidin-TRITC (1:1000) and DRAQ5 (1:8000). For PBMCs, after fixation and permeabilization, they were stained with 1:100 phalloidin-TRITC and 1:8000 DRAQ5 (#424101, Biolegend, San Diego, USA). Cells were analysed with BD LSR Fortessa (BD Biosciences, San Jose, USA). Analysis of flow cytometry data was performed with FlowJo (version 10.6.2., Becton, Dickinson and Company; 2019).

### Boyden chamber analysis

Fibroblasts were plated in a 24-well Transwell insert (Corning, Glendale, USA) at a density of 1 × 10^4^ in normal culture medium without fetal bovine serum. The bottom chamber was coated with collagen-I (#354236, Cornig) and filled with either serum free medium or 600 µl HAM-F12 culture medium containing 10% fetal bovine serum. After 24 hours, the top chamber was scraped with a cotton swab and 400 µl of 0.5% trypsin was added to the bottom chamber. Afterwards, 400 µl culture medium containing 1000× diluted Hoechst 3342 (#B2261, Sigma Aldrich) was added to the bottom chambers with the cells and immediately visualized with the EVOS™ XL Imaging System (ThermoFisher).

PBMCs were isolated from whole blood using Ficoll (#17-1440-02, GE Healthcare Hoevelaken, The Netherlands). Briefly, Ficoll was layered under whole blood mixed with one volume PBS and the whole was centrifuged for 20 min at 2400 rpm. PBMCs were collected, washed and frozen in 10% Dimethyl sulfoxide (DMSO) in fetal bovine serum. On the day of the assay, PBMCs were thawed and plated at a density of 50 000 cells in the upper part of 96-well transwell insert (# 3388, Cornig). PBMCs were allowed to migrate for 4 hours. Cells in both bottom and upper part of the transwell were fixed, permeabilized and stained with 1:100 phalloidin-TRITC and 1:8000 DRAQ5. Cells were counted using BD Fortessa LRS. The percentage of migrated cells was calculated by dividing cell count in the bottom chamber by the total number of cells (top and bottom chamber).

### Phenotypic overlap

To calculate the overlap between the phenotype of NAA80 patients to patients with other genetic diseases, we used PHRANK.^25^ As input we used the top-23 features with the highest occurrence ratio in our patients and the human phenotype ontology to disease list extracted from https://hpo.jax.org/app/download/annotation in July 2021.^26^ To extract phenotypic features of individuals with actin genetic variants, a literature search was performed in May 2020. Individual features in case reports were scored using human phenotype ontology scores and binary code (1= present, 0= not present) by two independent researchers. Occurrence ratios were calculated by dividing the percentage of individuals with a phenotypic feature by the number of genes associated with the phenotypic feature in human phenotype ontology. To avoid doubles (‘abnormality of the metopic ridge’ and ‘prominent metopic ride’), we used the human phenotype ontology code that was furthest up the tree.

### Statistical analysis

Differences in actin acetylation percentages were analysed using Student’s *t*-test (separate analysis for fibroblasts and PBMCs). The data are presented as mean ± SD.

Differences in Phalloidin median fluorescence intensity were analysed using Student’s *t*-test in case of two groups or ANOVA in case of three groups. The data are presented as mean ± SD. For the migration assays, ANOVA was used to compare groups. The data are presented as mean ± SD. Statistical analysis was carried out with Prism (GraphPad).

### Data availability

The authors declare that all data supporting the findings of this study are available within the paper and its supplementary information. Occurrence ratios for patients with variants in NAA80 or actin subtypes are added as Supplementary Table 4. The new variant described in this study was entered in ClinVar, accession number: VCV001301869.1

## Results

### Phenotypic description

**Figure 1.**
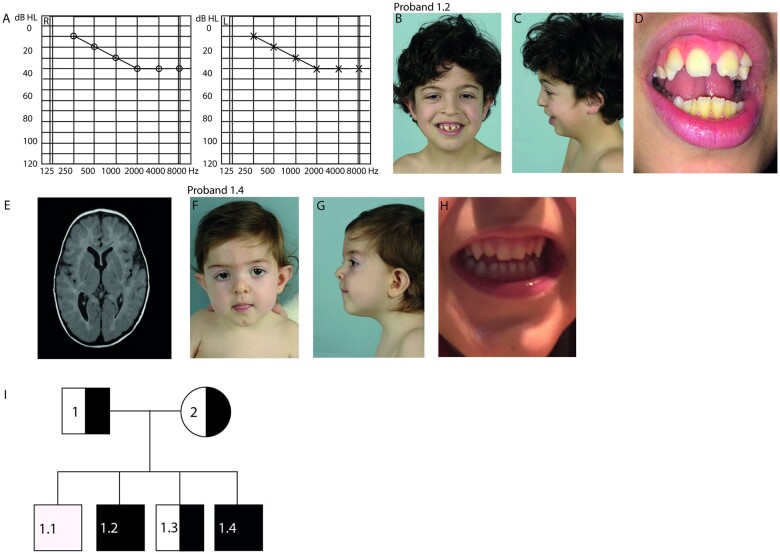
**NAA80 individuals present with craniofacial dysmorphisms, abnormal brain MRI and high**-**frequency hearing loss*.*** (A) Pure Tone Audiometry showing intensity measured in decibels (dB) represented on the *x*-axis and the frequency measured in Hertz (Hz) on the *y*-axis of the left ear (L) and right ear (R) of proband 1.2 showing high frequency hearing loss in the 2000–8000 kHz region. (B) Front view of proband 1.2 at the age of 7 years showing small upper lip, hypertelorism, abnormally shaped ears, ptosis and epicanthus fold. (C) Lateral view of proband 1.2 showing retrognathia. (D) Brain MRI image of proband 1.2 showing asymmetrical posterior horns. (E) Close-up of proband 1.2 showing diastema and peg-shaped lateral incisors. (F) Front view of proband 1.4 at the age of 1 year showing small upper lip, long philtrum, bulbous tip of the nose, ptosis, epicanthus fold, ocular hypertelorism, abnormally shaped ears. (G) Lateral view of proband 1.4. (H) Close-up of proband 1.4 showing peg-shaped lateral incisors. (I) Family pedigree of the NAA80 family. Black squares indicate affected male [homozygous for NAA80 c.389T>C, p.(Leu130Pro) variant]. Semi-black squares indicate that the individual is a carrier of a heterozygous NAA80 variant.

Proband 1.2 was born to Portuguese parents. After an unremarkable gestation and birth, he was diagnosed with bilateral high-frequency sensorineural hearing loss of 40 dB at the age of five weeks **(**Fig.  1A). Upon clinical examination, several dysmorphic features were noted, including hypertelorism, low posterior hair line, highly arched eyebrows, low-set protruding ears, small mouth, diastema, peg-shaped lateral incisors, disorganized implant of the toes and tapered fingers (Fig.  1B–D). In the following years, proband 1.2 exhibited periodic hypotonia, feeding difficulties, fatigue, swelling of his extremities and vomiting, which worsened during infections. He had behavioural disturbances, including rage and auto-mutilation, triggered by either loud noises or visual images. Additional phenotypic features included sleeping difficulties, loud snoring, gait ataxia and mild constipation requiring laxatives. Brain MRI revealed an enlarged right lateral ventricle system, a decreased amount of white matter volume and asymmetrical subcortical white matter lesions (Fig.  1E). Overall development was mildly delayed, with an estimated intelligence quotient of 79 at the age of three. Extensive etiological workup at our Hospital Centre for Evaluation of Developmental Delay, including clinical chemistry and metabolic screening in urine and plasma, did not reveal a cause for his clinical symptoms. Cardiac evaluation revealed mild systolic dysfunction, but no other abnormalities. SNP array revealed several small (<5 MB) and one large (8.8 MB) homozygous identity by descent regions (Supplementary Table 2). The large identity by descent region (8.8 MB) that was found in the patient exceeds the size of identity by descent regions found in outbred populations and suggests parents are distantly related.^27^^,^^28^ Parents originate from the same region in Portugal. Subsequent trio-exome sequencing allowed the identification of four genetic variants of unknown significance (Supplementary Table S3), from which the variant in the *NAA80* gene was considered most likely to be pathogenic: NC_000003.11: g.50334572A>G, c.389T>C, p.(L130P) (OMIM: 607073). Both parents were heterozygous for this variant.

Proband 1.4 was born 6 years later after an unremarkable pregnancy. His birth was complicated by respiratory distress due to meconium aspiration syndrome, necessitating mechanical ventilation for 24 hours. He had similar dysmorphic features as proband 1.2, including hypertelorism, low posterior hair line, highly arched eyebrows, low-set protruding ears, peg-shaped lateral incisors, small mouth and thin lips (Fig.  1E–G). In addition, a bilateral sensorineural hearing loss of 70 dB, most prominent in the high frequency 3 kHz area, was detected. He also experienced periodic hypotonia, feeding difficulties, mild constipation, sleeping difficulties and snoring. Additionally, he has multiple obstructive and central apnoeas per night (not resulting in hypoxia). Sanger sequencing confirmed homozygosity for the same variant in *NAA8*0: c.389T>C, p.(L130P) in proband 1.4, but not in healthy probands 1.1 and 1.3(Fig.  1I). Additionally, Sanger sequencing confirmed homozygosity for the same variant in *PLXNB1*: NC_000003.11: g.48460754T>C, c.2731A>G, p.(S911G). The PLXNB1 variant was not identified in the healthy siblings. With Sanger sequencing, we found the other genetic variants identified in proband 1.2 did not segregate within the family (Supplementary Table 3).

### Structural analysis of the NAA80 c.389T>C, p.(Leu130Pro) genetic variant

**Figure 2.**
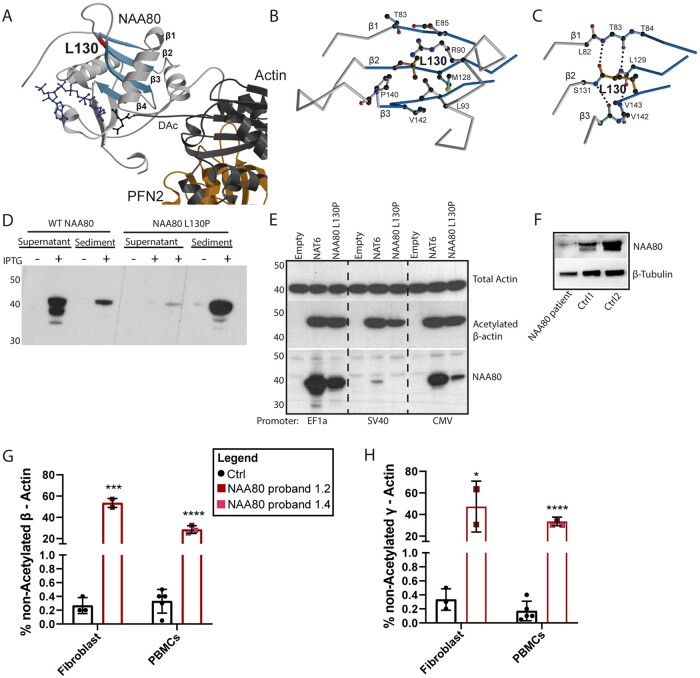
**The NAA80 c.389T>C, p.(Leu130Pro) variant decreases NAA80 stability and decreases actin acetylation.** (A) Crystal structure of NAA80 bound to the actin–profilin complex. NAA80 is shown in grey with β-strands 1–4 highlighted in blue and Leu130 in red. Actin is shown in dark grey and profilin in orange. Coenzyme A (CoA) in blue and the acetylated N-terminal aspartate (DAc) in dark grey are in ball-and-stick representation. The colour-coding is used throughout the figure. (B) Detailed view of the local environment of the side chain of Leu130. The peptide chain is shown as Cα-trace with selected side chains in ball-and-stick representation. (C) Hydrogen network of the backbone surrounding Leu130. With the exception of Leu130, residues are shown without sidechains as glycine. Dotted lines represent hydrogen bonds. (D) Western blot analysis of the expression of wild-type (WT) and mutant NAA80 in *Escherichia coli*. Induction of the expression with IPTG leads to the appearance of a protein that is mainly present in the supernatant (SN) of bacterial extracts in the case of the wild-type protein and in the sediment in the case of the mutant. (E) Western blot of HAP1 NAA80 knockout cells infected with lentiviral vectors driving the expression of WT NAA80, Leu130Pro NAA80 and no protein (empty). Mutant NAA80 generates decreased levels of NAA80 protein, and with the weakest promoter, detectably decreased acetylated β-actin levels. One representative experiment out of two is shown. (F) Western blot showing severely decreased NAA80 protein expression in fibroblasts of proband 1.2 compared to two healthy donors, β-tubulin was used as a housekeeper. One representative experiment out of two is shown. (G) Bar graph representing the proportions of ‘endogenously’ unacetylated β-actin (expressed as percentage of acetylated + unacetylated β-actin) of healthy controls (black) and probands 1.2 and 1.4 (red). Two independent experiments were performed of both the fibroblasts (proband 1.2) and the PBMCs (both probands), and each cell type was compared to three different healthy control samples (combined data from two experiments). Coloured squares represent mean values of all technical replicates, bars represent mean values of healthy controls (Ctrl) or individuals ± SD. *P < 0.05, ***P < 0.001 (Student’s *t*-test). Acetylation of the N-terminus of actins was determined by /MS/MS analysis of tryptic peptides as described in the ‘Materials and Methods’ section. (H) Bar graph represents the percentage of unacetylated γ-actin in three healthy controls (black) and proband 1.2 and 1.4 (red). Two independent experiments were performed of both the fibroblasts (proband 1.2 only) and the PBMCs (both patients), and each cell type was compared to three different healthy control samples (two experiments). Coloured squares represent technical replicates, bars represent mean per donor ± SD. *P < 0.05, ***P < 0.001 (Student’s *t*-test).

NAA80 includes an N-terminal extension, followed by a catalytic domain (residues 78-220), a proline-rich loop and a stretch of residues also belonging to the catalytic domain.^7^ Leu130 is localized at the end of β-strand β1, which is part of a central β-sheet of the catalytic domain (Fig.  2A). The side chain of Leu130 points into a hydrophobic environment (Fig.  2B). The backbone of Leu130 is involved in the formation of the hydrogen bond network of the central β-sheet, with direct hydrogen bonds to Thr83 in β1 (Fig.  2C). Replacement by a proline is expected to disturb the hydrogen bond network, in particular of the nitrogen atom in the peptide bond. Furthermore, adaptations would be required due to the sterical demands of the proline ring. These effects are expected to weaken and disturb the local fold of the protein.

### The NAA80 c.389T>C, p.(Leu130Pro) variant causes defective protein folding, resulting in limited protein availability

The wild-type and the mutant NAA80 protein with a C-terminal poly-His tag were expressed in *E.coli*, and bacterial extracts were centrifuged and analysed by sodium dodecyl sulphate polyacrylamide gel electrophoresis and western blot. Mutant protein was predominantly insoluble, contrasting with wild-type protein, which was essentially soluble, with only a very small proportion in the sediment (Fig.  2D). These findings align with the prediction that the *NAA80* variant induces defective protein folding.

To measure the impact of the *NAA80* genetic variant on protein levels in mammalian cells, we infected NAA80 knockout HAP1 cells with constructs expressing mutant or wild-type NAA80 cDNA with promoters of different strengths, EF1A being the strongest and SV40 being the weakest promoter. Lower protein levels of mutant NAA80 compared with healthy donors were observed with all promoters (Fig.  2E). Similarly, in NAA80 individual fibroblasts, NAA80 protein levels were lower compared to healthy controls (Fig.  2F). Use of the weakest promoter resulted in partial restoration of actin acetylation, as determined by western blotting with antibodies specific for the acetylated form of beta actin, while stronger promoters led to maximal or near maximal acetylation, indicating that the mutated protein still shows residual activity (Fig.  2E). In addition, we found that expression of low levels of wild-type NAA80 in HAP1 knockout cells was already sufficient to reach maximal or near-maximal acetylation (Fig.  2E). It should be noted that the western blot approach used in this experiment does not allow precise quantification of the level of acetylation.

### The NAA80 c.389T>C, p.(Leu130Pro) genetic variant results in decreased, but not absent, actin acetylation

To accurately determine the levels of acetylated actin, the level of N-terminal acetylation of β- and γ-actin in fibroblasts and PBMCs from NAA80 individuals was analysed by mass spectrometry (Fig.  2G and H). In healthy donors, ∼0.5% of beta and gamma-actins were not acetylated. However, for the NAA80 individuals, 25–65% of beta and gamma-actins were not acetylated, depending on the cell type and the type of cytoplasmic actin (Fig.  2G and H).

### NAA80 individual fibroblasts show increased rates of polymerized actin, increased filopodia counts and increased cellular migration

**Figure 3.**
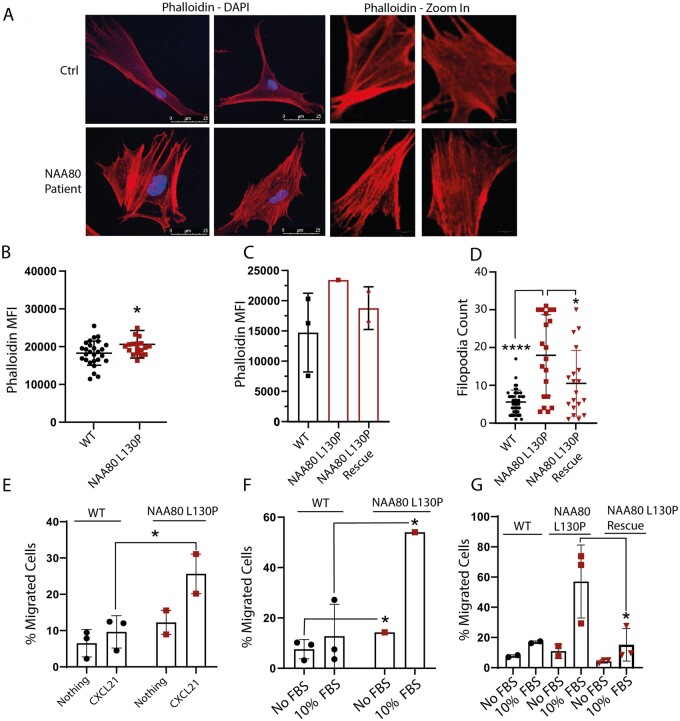
**NAA80 variants lead to increased actin polymerization, filopodia count and cellular migration.** (A) Microscopy images of healthy Ctrl fibroblasts (upper panel) and NAA80 individual fibroblasts (lower panel) . NAA80 individual fibroblasts show increased filopodia counts and altered morphology of the polymerized actin network. (B) Phalloidin mean fluorescence intensity of healthy controls (*N* = 3, nine technical replicates) and NAA80 patient PBMCs (*N* = 2, nine technical replicates). Dots represent the mean phalloidin staining (median fluorescence intensity) of each replicate, the line represents the mean of all replicates ±SD ****P < 0.001 (Student’s *t*-test). (C) Phalloidin median fluorescence intensity of healthy controls (*N* = 3, one technical replicate each) and NAA80 individual fibroblasts (*N* = 1, one technical replicate) and fibroblasts of proband 1.2 expressing WT NAA80 with CMV or SV40 promoter (*N* = 2, both one replicate). Bar represents mean fluorescence intensity of the phalloidin staining ±SD. (D) Filopodia count of healthy control (*N* = 3) and individual fibroblasts (*N* = 1) transfected with an empty vector and SV40 promoter, and individual fibroblasts with NAA80 WT expressing plasmid with SV40 promoter (*N* = 1). Dots represent filopodia count per cell, lines represent mean filopodia count of all cells ±SD. *P < 0.05, ****P < 0.001 (ANOVA). (E) Cellular migration measured with the Boyden chamber assay of PBMCs with normal culture medium (nothing) or CXCL21 enriched culture medium (CXCL21) in the bottom chamber. Dots and squares represent mean migration of three technical replicates, bars represent mean migration of healthy controls combined (*N* = 3) or NAA80 individuals (*N* = 2) ±SD. *P < 0.05 (Student’s *t*-test). (F) Cellular migration of control and NAA80 individual fibroblasts, measured with the Boyden chamber assay, with normal culture medium without (No FBS) or with (10% FBS) enriched culture medium in the bottom chamber. Dots or squares represent mean migration of three technical replicates, bars represent mean migration percentage of three donors ±SD. *P < 0.05 (Student’s *t*-test). (G) Cellular migration of healthy control (*N* = 2) and NAA80 individual fibroblasts transduced with an empty vector (*N* = 1, three technical replicates) and NAA80 individual fibroblasts transduced with a NAA80 wild-type expressing vector (*N* = 1, three technical replicates). Bars represent mean migration per condition ±SD. *P < 0.05.

The depolymerization rate for acetylated actin is approximately 2-fold higher than for non-acetylated actin.^9^ Therefore, we hypothesized that the higher proportion of non-acetylated actin in NAA80 individuals would decrease the overall depolymerization rate, resulting in increased levels of polymerized actin. Indeed, NAA80 individual PBMCs and fibroblasts displayed increased phalloidin staining, corresponding with increased levels of polymerized actin (Fig.  3A and B). Expressing wild-type NAA80 with the SV40 promoter in NAA80 individual fibroblasts normalized phalloidin staining intensity (Supplementary Fig. 2 and Fig.  3C).

Since HAP1 NAA80 knockout cells showed increased filopodia formation and cellular migration^9^, filopodia counts and migration dynamics were quantified. In NAA80 individual fibroblasts, increased filopodia counts were observed (Fig.  3D). The introduction of wild-type NAA80 protein with the SV40 promoter lowered filopodia counts to the level observed in healthy controls (Fig.  3D).

Cellular migration in response to chemotactic stimuli was tested using the *Boyden* chamber assay. Both fibroblasts and PBMCs of NAA80 individuals showed increased movement in response to chemotactic stimuli (Fig.  3E and F). In fibroblasts, expression of NAA80 wild-type with the SV40 promoter protein lowered migration levels to the level observed in healthy controls (Fig.  3G).

Together, these results indicate that the NAA80 genetic variant results in decreased NAA80 protein levels and acetylation activity causing a distinct cellular phenotype, similar to that observed in NAA80 HAP1 knockout cell lines.

### Individuals with the NAA80 c.389T>C, p.(Leu130Pro) variant show significant phenotypic overlap with individuals with pathogenic variants in ACTB and ACTG1

To relate our molecular findings to the clinical phenotype, NAA80 individuals were compared with individuals harbouring genetic variants in actin genes. For comparison, we used occurrence ratios that grant more weight to specific features (e.g. high-frequency hearing loss) than to non-specific features (developmental delay) by dividing the percentage of individuals presenting with a phenotypic feature by the total number of genes associated with that phenotypic feature.^29^ With this approach, one can define overlap for rarely seen phenotypic features, which may point in the direction of shared pathophysiology.

**Figure 4.**
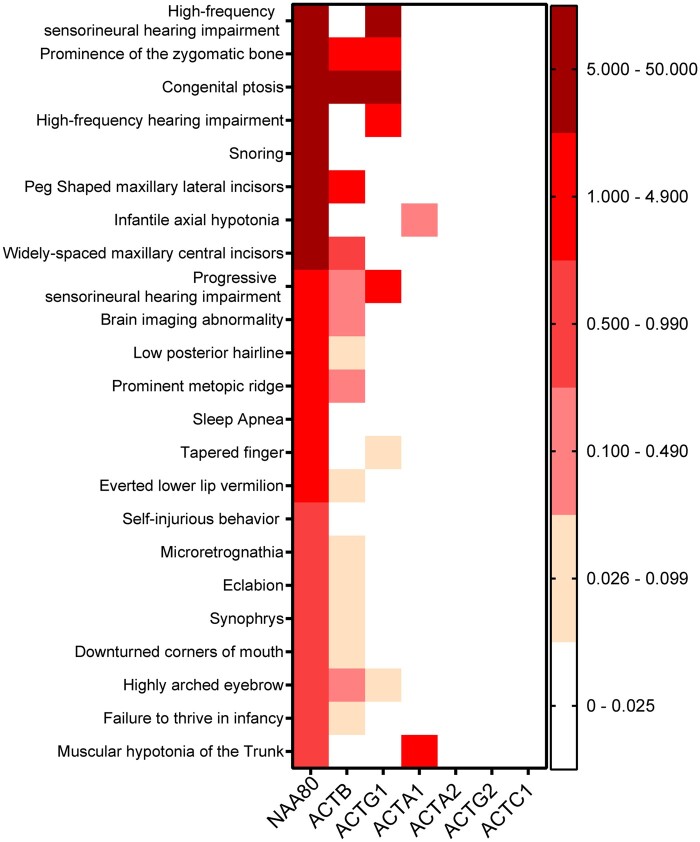
**The most specific phenotypic features of NAA80 individuals overlap with those of individuals with ACTB, ACTG1 and ACTA variants.** Colour coded occurrence ratios of the most specific phenotypic features of NAA80 individuals with corresponding occurrence ratios of individuals with other actin genetic variants. ACTG1 = actin-γ; ACTB = actin-β; ACTA1 = muscle α-actin; ACTA2 = smooth muscle actin-α; ACTG2 = gamma-enteric smooth muscle actin; ACTC1 = cardiac, muscle alpha actin.

To calculate the similarity between phenotypes, we employed the PHRANK^25^ tool, using the top-23 phenotypic features with the highest occurrence ratio (depicted in Fig.  4), comparing it to the phenotype of each actin gene. We found that Baraitser-Winter (OMIM #243310, resulting from ACTB and ACTG1 mutations) was in the top three of diseases that most resembled the NAA80 phenotype, together with Rubinstein–Taybi syndrome (OMIM #180849) and Wolf–Hirschhorn syndrome (OMIM #194190).

Strikingly, comparing NAA80 patients to ACTB patients gave a similarity score of 60.29, and 30.37 when compared to patients with ACTG1 mutations (Fig.  4). The overlap with ACTA1 was only modest: NAA80 individuals exhibited moderate axial hypotonia and proximal muscle weakness, while ACTA1 individuals have overt nemaline myopathy (similarity score of 20). The similarity scores of NAA80 and ACTG2, ACTC1 and ACTA2 were negligible (1.00, 0.00 and 0.00, respectively). While ACTG2 individuals show intestinal pseudo-obstruction, only mild constipation is seen in NAA80 individuals. Similarly, the ventricular dysfunction seen in NAA80 individuals could reflect a minor ACTC1 dysfunction. Together, these results show that NAA80 individuals resemble the phenotype seen in individuals with ACTG1, ACTB and ACTA1 mutations, but not in individuals with ACTG2, ACTC1 and ACTA2 mutations.

## Discussion

Until now, the consequences of N-terminal actin acetylation have remained largely unexplored *in vivo*. Here, we describe a new syndrome characterized by high-frequency hearing loss, developmental delay and muscle weakness, caused by homozygous NAA80 c.389T>C, p.(Leu130Pro) variants. We provide evidence that the c.389T>C, p.(Leu130Pro) variant destabilizes NAA80, resulting in decreased, but not absent, enzymatic activity and consequently decreased actin acetylation. This resulted in increased filopodia formation, increased cellular movement and increased levels of polymerized actin. Together, our results underscore the importance of NAA80-mediated actin acetylation *in vivo.*

The shared clinical features of NAA80 individuals and ACTB/ACTG1 individuals, for example hearing loss, suggest a similar underlying pathophysiological mechanism. Most individuals with ACTB/ACTG1 variants that exhibit hearing loss have normal actin abundancy, but show altered actin dynamics *in vitro*, suggesting that altered actin dynamics rather than absolute actin concentrations contribute to hearing loss.^30–33^ Auditory hair cells consist of a particularly stable actin core with new actin incorporation only at the distal tips, hence requiring controlled actin dynamics.^34^ To facilitate a stable actin core and controlled incorporation of F-actin, actin stabilizing proteins such as PLS1, FSCN2 and XIRP2 are highly expressed in the inner ear.^35–38^ For example, loss of plastin-1 (PLS1) function, which limits actin depolymerization to prevent thinning of the actin bundle, results in hearing loss.^35^^,^^38^^,^^39^ As NAA80-mediated actin acetylation results in altered actin dynamics,^9^ it is likely that normal cytoskeletal architecture is disrupted in NAA80 patients, causing hearing loss.

We found increased filopodia counts in NAA80 patient fibroblasts. In the brain, filopodia are precursors for dendritic spines, that eventually form excitatory synapses.^40–43^ Disturbances in filopodia formation can lead to severe neuronal migration disorders, illustrated by the observation that genetic variants in actin remodelling genes, including *PAFAH1B1*, show decreased filopodia formation and decreased neuronal migration, resulting in autism spectrum disorder, developmental delay and epileptic seizures.^44–46^ Likewise, individuals with ACTB and ACTG1 variants and Baraitser-Winter syndrome frequently present with neuronal migration disorders like lissencephaly and pachygyria resulting in mild intellectual disability and seizures.^47^ However, the consequences of *increased* filopodia formation, which occurs in our patients, have not yet been established. Speculatively, excessive filopodia numbers could lead to uncontrolled formation of excitatory synapses that might relate to the NAA80 individuals’ hyperarousal, behavioural disturbances and mild intellectual disability. Additionally, the asymmetry seen in the lateral ventricles of proband 1.2 could reflect a minor neuronal migration disorder.

Even though NAA80 is predicted to have similar affinity for all six actin isoforms, there was no overt clinical overlap between NAA80 individuals and individuals with ACTA2, ACTG2 and ACTC1 genetic variants. Some of these features might not have yet developed in NAA80 individuals, as ACTA2- and ACTC1-related features often do not manifest until the second or third decade of life.^48–53^ However, individuals with ACTG2 genetic variants are usually identified early in life, with severe chronic intestinal pseudo obstruction.^54^^,^^55^ Alternatively, one could speculate that the constipation and mild ventricular dysfunction seen in NAA80 individuals reflect a minor ACTG2 and ACTC1 dysfunction, in a similar way as the mild myopathy of NAA80 individuals reflects a minor form of the severe, lethal myopathy seen in ACTA1 individuals. The latter would imply that NAA80-mediated acetylation might have a more modest role in the function of muscle actin isoforms, which might relate to the more stable nature of actin isoforms within muscle, making them less vulnerable to flawed actin dynamics.

**Figure 5.**
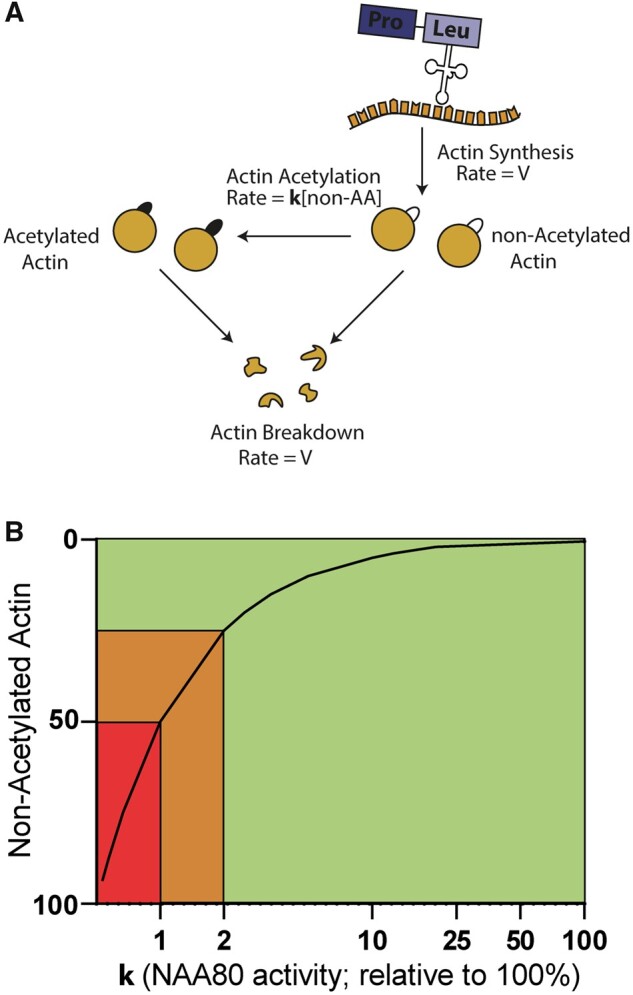
**Graphical model showing the NAA80 activity (*x*-axis) versus the amount of substrate (non-acetylated actin, *y*-axis).** (A) Under steady-state conditions, actin is synthesized, acetylated and degraded. For the sake of simplicity, we assume that the degradation of acetylated and non-acetylated actins proceed with the same rate constant. If we assume that the rate of acetylation is proportional to the non-acetylated actin (non-AA) concentration, *V* = k [non-AA], where **k** is the constant rate of the reaction catalysed by NAA80. The concentration of non-AA is therefore equal to *V*/*k*. (B) An increase in the proportion of non-acetylated actin from 0.5% (normal value) to 25% or 50% may be due to a reduction in the NAA80 activity of 50- and 100-fold, respectively (i.e. from a value of 100% to 2 or 1% approximately). Therefore, the reduced NAA80 activity caused by NAA80 genetic variants in our individuals falls within a critical range (orange area) that is disease-causing but not lethal. Less than 1% NAA80 activity is thought to be lethal (red area). Over 2% of NAA80 residual activity probably does not result in overt clinical symptoms (green area). Note that all percentages mentioned are based on estimates. Note that the *x*-axis has a logarithmic scale.

Surprisingly, the severely reduced NAA80 availability only resulted in a modest (≈50%) decrease of actin acetylation. The relationship between NAA80 activity and substrate availability (unacetylated actins) can be studied with a simplified model, in which we assume that NAA80 activity (*k*) is proportional to the concentration of non-acetylated actin (non-AA), i.e. that it is equal to k•[non-AA] (Fig. 5). If we consider that the rate of non-acetylated actin formation and degradation (*V*) is the same in control cells and in cells with the NAA80 variant, a 50-fold or 100-fold increase in the concentration of non-acetylated actin (i.e. 25% or 50% in individual cells versus 0.5% in controls) can be accounted for by a 50-fold or 100-fold decrease in the value of *k*. Thus, ∼50% actin acetylation agrees with extremely low, but not absent, residual NAA80 activity, estimated in the range of 1–2% of normal activity.

If NAA80 activity were to drop below the level observed in NAA80 individuals, over 50% of actins would be affected. This level is probably lethal, since bi-allelic actin variants—affecting over 50% of actins—do not exist. In contrast, if NAA80 genetic variants resulted in *more* residual activity than is seen in NAA80 individuals, actin acetylation still reaches ∼85%, a level that will probably not cause an overt clinical phenotype. Thus, it is likely that only a narrow range of residual NAA80 catalytic activity is tolerated *and* disease causing. There is a slim chance that NAA80 genetic variants cause actin acetylation levels that fall exactly within this critical range, which explains why we could not identify additional individuals with NAA80 genetic variants, despite extensive searches in GeneMatcher and population databases.

In conclusion, we here demonstrate that patients with NAA80 genetic variants are pathogenic and result in actin acetylation defects. Since NAA80 activity is directly linked to substrate availability, only a specific range of NAA80 catalytic activity is tolerated and disease causing, explaining why bi-allelic NAA80 genetic variants are extremely rare. The significant clinical overlap between individuals with NAA80 and ACTB/ACTG1 variants suggests a more prominent role for NAA80 for cytoplasmic actin isoforms. These insights underscore the importance of fine-tuned actin dynamics by actin acetylation for human health.

## Supplementary material

Supplementary material is available at Brain Communications online.

## Supplementary Material

fcab256_Supplementary_Data

## Abbreviations

AGC =: automatic gain control
BCA =: bicinchoninic acid
FBS =: fetal bovine serum
HCD =: high energy collision dissociation
HEK293 =: human embryonic kidney 293
HPO =: human phenotype ontology
IQ =: intelligence quotient
KCl =: kalium chloride
KO =: knockout
MAF =: minor allele frequency
MFI =: median fluorescence intensity
non-AA =: non-acetylated actin
PBMCs =: peripheral blood mononuclear cells
PMSF =: phenylmethylsulfonyl fluoride
SNP =: single-nucleotide polymorphism

## References

[1] Pollard TD , Cooper JA. Actin, a central player in cell shape and movement. Science. 2009;326(5957):1208–1212. 19965462 10.1126/science.1175862 PMC3677050

[2] Bunnell TM , Burbach BJ, Shimizu Y, Ervasti JM. β-Actin specifically controls cell growth, migration, and the G-actin pool. Mol Biol Cell. 2011;22(21):4047–4058. 21900491 10.1091/mbc.E11-06-0582 PMC3204067

[3] Belyantseva IA , Perrin BJ, Sonnemann KJ, et al γ-Actin is required for cytoskeletal maintenance but not development. Proc Natl Acad Sci USA. 2009;106(24):9703–9708. 19497859 10.1073/pnas.0900221106 PMC2701000

[4] Vandekerckhove J , Bugaisky G, Buckingham M. Simultaneous expression of skeletal muscle and heart actin proteins in various striated muscle tissues and cells. J Biol Chem. 1986;261(4):1838–1843. 3944112

[5] Hoock TC , Newcomb PM, Herman IM. β Actin and its mRNA are localized at the plasma membrane and the regions of moving cytoplasm during the cellular response to injury. J Cell Biol. 1991;112(4):653–664. 1993736 10.1083/jcb.112.4.653 PMC2288855

[6] Micheva KD , Vallée A, Beaulieu C, Herman IM, Leclerc N. Β-Actin is confined to structures having high capacity of remodelling in developing and adult rat cerebellum. Eur J Neurosci. 1998;10(12):3785–3798. 9875357 10.1046/j.1460-9568.1998.00391.x

[7] Rebowski G , Boczkowska M, Drazic A. Mechanism of actin N-terminal acetylation. Sci Adv. 2020;6(15):eaay8793. 10.1126/sciadv.aay8793 PMC7141826 32284999

[8] Wiame E , Tahay G, Tyteca D, et al NAT6 acetylates the N-terminus of different forms of actin. FEBS J. 2018;285(17):3299–3316. 30028079 10.1111/febs.14605

[9] Drazic A , Aksnes H, Marie M, et al NAA80 is actin’s N-terminal acetyltransferase and regulates cytoskeleton assembly and cell motility. Proc Natl Acad Sci USA. 2018;115(17):4399–4404. 29581253 10.1073/pnas.1718336115 PMC5924898

[10] Goris M , Magin RS, Foyn H, et al Structural determinants and cellular environment define processed actin as the sole substrate of the N-terminal acetyltransferase NAA80. Proc Natl Acad Sci USA. 2018;115(17):4405–4410. 29581307 10.1073/pnas.1719251115 PMC5924903

[11] Aksnes H , Drazic A, Marie M, Arnesen T. First things first: vital protein marks by N-terminal acetyltransferases. Trends Biochem Sci. 2016;41(9):746–760. 27498224 10.1016/j.tibs.2016.07.005

[12] Ree R , Kind L, Kaziales A, et al PFN2 and NAA80 cooperate to efficiently acetylate the N-terminus of actin. J Biol Chem. 2020;295(49):16713–16731. 32978259 10.1074/jbc.RA120.015468 PMC7864067

[13] Varland S , Vandekerckhove J, Drazic A. Actin post-translational modifications: the cinderella of cytoskeletal control. Trends Biochem Sci. 2019;44(6):502–516. 30611609 10.1016/j.tibs.2018.11.010

[14] Donnelly P , Green ED, Knoppers BM, et al The 1000 Genomes Project Consortium. A global reference for human genetic variation. Nature. 2015;526(7571):68–74. 26432245 10.1038/nature15393 PMC4750478

[15] Zerbino DR , Achuthan P, Akanni W, et al Ensembl 2018. Nucleic Acids Res. 2018;46(D1):D754–D761. 29155950 10.1093/nar/gkx1098 PMC5753206

[16] Lek M , Karczewski K, Minikel E, et al Analysis of protein-coding genetic variation in 60,706 humans. Nature. 2016;536:285–291 10.1038/nature19057 PMC5018207 27535533

[17] Karczewski KJ , Francioli LC, Tiao G, et al The mutational constraint spectrum quantified from variation in 141,456 humans [Published online 2019:531210]. *bioRxiv*. doi:10.1101/531210. 10.1038/s41586-020-2308-7 PMC7334197 32461654

[18] Schomburg D , Reichelt J. BRAGI: a comprehensive protein modeling program system. J Mol Graph. 1988;6(3):161–165.

[19] Kraulis PJ. MOLSCRIPT. A program to produce both detailed and schematic plots of protein structures. J Appl Crystallogr. 1991;24(5):946–950.

[20] Merritt EA , Murphy ME, Raster3D version-2.0 - A program for photorealistic molecular graphics. Acta Crystallogr D Biol Crystallogr. 1994;50(Pt 6):869–873. 15299354 10.1107/S0907444994006396

[21] Ury B , Potelle S, Caligiore F, et al. The promiscuous binding pocket of SLC35A1 ensures redundant transport of CDP-ribitol to the Golgi. J Biol Chem. 2021;296:100789. 34015330 10.1016/j.jbc.2021.100789 PMC8192872

[22] Dewulf JP , Wiame E, Dorboz I, et al SLC13A3 variants cause acute reversible leukoencephalopathy and α-ketoglutarate accumulation. Ann Neurol. 2019;85(3):385–395. 30635937 10.1002/ana.25412

[23] Pino LK , Searle BC, Bollinger JG, Nunn B, MacLean B, MacCoss MJ. The Skyline ecosystem: informatics for quantitative mass spectrometry proteomics. Mass Spectrom Rev. 2020;39(3):229–244. 28691345 10.1002/mas.21540 PMC5799042

[24] Jacquemet G , Paatero I, Carisey AF, et al FiloQuant reveals increased filopodia density during breast cancer progression. J Cell Biol. 2017;216(10):3387–3403. 28765364 10.1083/jcb.201704045 PMC5626550

[25] Jagadeesh K , Birgmeier J, Guturu H, et al Phrank measures phenotype sets similarity to greatly improve Mendelian diagnostic disease prioritization. Genet Med. 2019;21:464–470. 29997393 10.1038/s41436-018-0072-y

[26] Köhler S , Gargano M, Matentzoglu N, et al The human phenotype ontology. Nucleic Acids Res. 2021;49(D1):D1207–D1217. 33264411 10.1093/nar/gkaa1043 PMC7778952

[27] Palamara PF , Lencz T, Darvasi A, Pe’er I. Length distributions of identity by descent reveal fine-scale demographic history. Am J Hum Genet. 2012;91(5):809-822. 23103233 10.1016/j.ajhg.2012.08.030 PMC3487132

[28] Shetty A , O’Connell J, Mitchell B, O’Connor T. Rare variant enriched identity-by-descent enables the detection of distant relatedness and older divergence between populations [Published online May 7, 2020]. *bioRxiv*. doi:10.1101/2020.05.05.079541.

[29] Haijes HA , Jaeken J, van Hasselt PM. Hypothesis: determining phenotypic specificity facilitates understanding of pathophysiology in rare genetic disorders. J Inherit Metab Dis. 2020;43(4):701–711. 31804708 10.1002/jimd.12201 PMC7383723

[30] Bryan KE , Wen KK, Zhu M, et al Effects of human deafness γ-actin mutations (DFNA20/26) on actin function. J Biol Chem. 2006;281(29):20129–20139. 16690605 10.1074/jbc.M601514200

[31] Zhu M , Yang T, Wei S, et al Mutations in the γ-actin gene (ACTG1) are associated with dominant progressive deafness (DFNA20/26). Am J Hum Genet. 2003;73(5):1082–1091. 13680526 10.1086/379286 PMC1180488

[32] Vedula P , Kashina A. The makings of the ‘actin code’: regulation of actin’s biological function at the amino acid and nucleotide level. J Cell Sci. 2018;131(9):jcs215509. 10.1242/jcs.215509 PMC5992587 29739859

[33] Rivière JB , Van Bon BWM, Hoischen A, et al De novo mutations in the actin genes ACTB and ACTG1 cause Baraitser-Winter syndrome. Nat Genet. 2012;44(4):440–444. 22366783 10.1038/ng.1091 PMC3677859

[34] Drummond MC , Barzik M, Bird JE, et al Live-cell imaging of actin dynamics reveals mechanisms of stereocilia length regulation in the inner ear. Nat Commun. 2015;6:doi:10.1038/ncomms7873 10.1038/ncomms7873 PMC4411292 25898120

[35] Roy P , Perrin BJ. The stable actin core of mechanosensory stereocilia features continuous turnover of actin cross-linkers. Mol Biol Cell. 2018;29(15):1856–1865. 29874122 10.1091/mbc.E18-03-0196 PMC6085822

[36] Perrin BJ , Strandjord DM, Narayanan P, et al Beta-actin and fascin-2 cooperate to maintain stereocilia length. J Neurosci. 2013;33(19):8114–8121. 23658152 10.1523/JNEUROSCI.0238-13.2013 PMC3718021

[37] Scheffer DI , Zhang D-S, Shen J, et al XIRP2, an actin-binding protein essential for inner ear hair-cell stereocilia. Cell Rep. 2015;10(11):1811–1818. 25772365 10.1016/j.celrep.2015.02.042 PMC4376604

[38] Taylor R , Bullen A, Johnson SL, et al Absence of plastin 1 causes abnormal maintenance of hair cell stereocilia and a moderate form of hearing loss in mice. Hum Mol Genet. 2015;24(1):37–49. 25124451 10.1093/hmg/ddu417 PMC4262491

[39] Morgan A , Koboldt DC, Barrie ES, et al Mutations in PLS1, encoding fimbrin, cause autosomal dominant nonsyndromic hearing loss. Hum Mutat. 2019;40(12):2286–2295. 31397523 10.1002/humu.23891

[40] Marrs GS , Green SH, Dailey ME. Rapid formation and remodeling of postsynaptic densities in developing dendrites. Nat Neurosci. 2001;4(10):1006–1013. 11574832 10.1038/nn717

[41] Ziv NE , Smith SJ. Evidence for a role of dendritic filopodia in synaptogenesis and spine formation. Neuron. 1996;17(1):91–102. 8755481 10.1016/s0896-6273(00)80283-4

[42] Vaugn JE. Fine structure of synaptogenesis in the vertebrate central nervous system. Synapse. 1989;3(3):255–285. 2655146 10.1002/syn.890030312

[43] Niell CM , Meyer MP, Smith SJ. In vivo imaging of synapse formation on a growing dendritic arbor. Nat Neurosci. 2004;7(3):254–260. 14758365 10.1038/nn1191

[44] Cardoso C , Leventer RJ, Dowling JJ, et al Clinical and molecular basis of classical lissencephaly: mutations in the LIS1 gene (PAFAH1B1). Hum Mutat. 2002;19(1):4–15. 11754098 10.1002/humu.10028

[45] Kholmanskikh SS , Koeller HB, Wynshaw-Boris A et al Calcium-dependent interaction of Lis1 with IQGAP1 and Cdc42 promotes neuronal motility. Nat Neurosci. 2006;9(1):50–57. 16369480 10.1038/nn1619

[46] Kholmanskikh SS , Dobrin JS, Wynshaw-Boris A, Letourneau PC, Ross ME. Disregulated RhoGTPases and actin cytoskeleton contribute to the migration defect in Lis1-deficient neurons. J Neurosci. 2003;23(25):8673–8681. 14507966 10.1523/JNEUROSCI.23-25-08673.2003 PMC6740411

[47] Verloes A , Di Donato N, Masliah-Planchon J, et al Baraitser-Winter cerebrofrontofacial syndrome: delineation of the spectrum in 42 cases. Eur J Hum Genet. 2015;23(3):292–301. 25052316 10.1038/ejhg.2014.95 PMC4326722

[48] Guo DC , Papke CL, Tran-Fadulu V, et al Mutations in smooth muscle alpha-actin (ACTA2) cause coronary artery disease, stroke, and Moyamoya disease, along with thoracic aortic disease. Am J Hum Genet. 2009;84(5):617–627. 19409525 10.1016/j.ajhg.2009.04.007 PMC2680995

[49] Guo DC , Pannu H, Tran-Fadulu V, et al Mutations in smooth muscle alpha-actin (ACTA2) lead to thoracic aortic aneurysms and dissections. Nat Genet. 2007;39(12):1488–1493. 17994018 10.1038/ng.2007.6

[50] Yuan SM. α-Smooth muscle actin and ACTA2 gene expressions in vasculopathies. Brazilian J Cardiovasc Surg. 2015;30(6):644–649. 10.5935/1678-9741.20150081 PMC4762557 26934405

[51] Monserrat L , Hermida-Prieto M, Fernandez X, et al Mutation in the alpha-cardiac actin gene associated with apical hypertrophic cardiomyopathy, left ventricular non-compaction, and septal defects. Eur Heart J. 2007;28(16):1953–1961. 17611253 10.1093/eurheartj/ehm239

[52] Olson TM , Doan TP, Kishimoto NY, Whitby FG, Ackerman MJ, Fananapazir L. Inherited and de novo mutations in the cardiac actin gene cause hypertrophic cardiomyopathy. J Mol Cell Cardiol. 2000;32(9):1687–1694. 10966831 10.1006/jmcc.2000.1204

[53] Mogensen J , Klausen IC, Pedersen AK, et al Αlpha-cardiac actin is a novel disease gene in familial hypertrophic cardiomyopathy. J Clin Invest. 1999;103(10):R39–R43. 10330430 10.1172/JCI6460 PMC408458

[54] Lehtonen HJ , Sipponen T, Tojkander S, et al Segregation of a missense variant in enteric smooth muscle actin γ-2 with autosomal dominant familial visceral myopathy. Gastroenterology. 2012;143(6):1482–1491.e3. 22960657 10.1053/j.gastro.2012.08.045

[55] Wangler MF , Gonzaga-Jauregui C, Gambin T, et al Baylor-Hopkins Center for Mendelian Genomics. Heterozygous de novo and inherited mutations in the smooth muscle actin (ACTG2) gene underlie megacystis-microcolon-intestinal hypoperistalsis syndrome. PLoS Genet. 2014;10(3):e1004258. 24676022 10.1371/journal.pgen.1004258 PMC3967950

